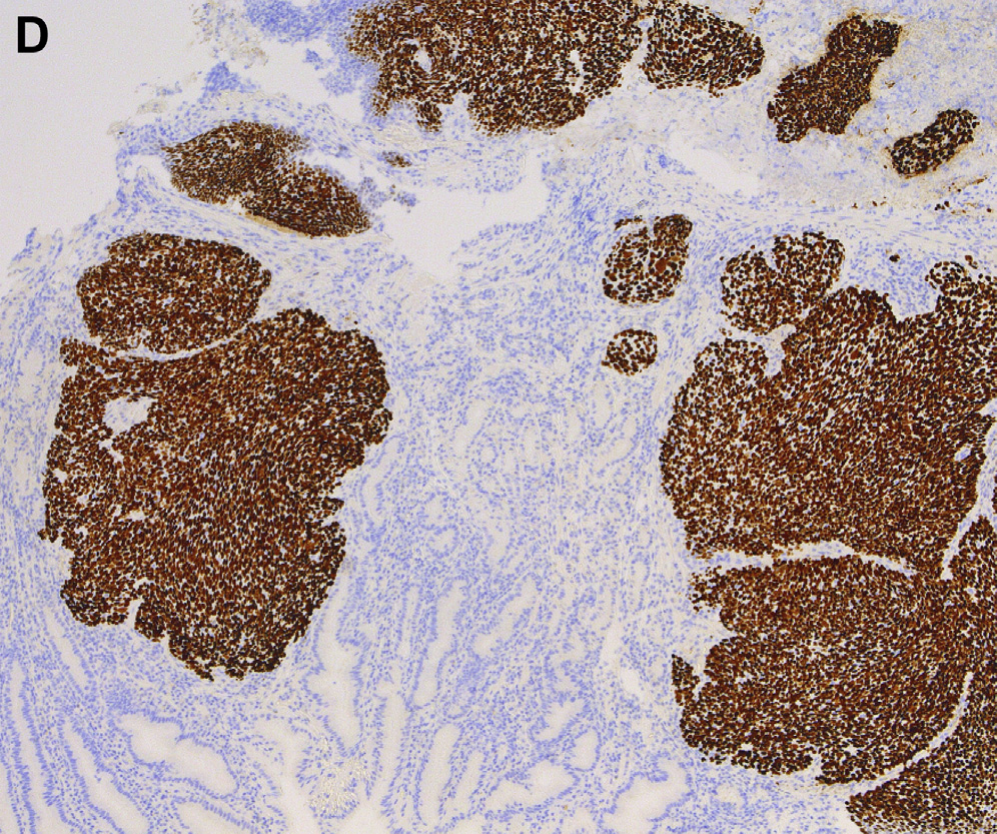# A Rare Finding of Gastric Metastasis From Tonsillar Squamous Cell Carcinoma

**DOI:** 10.1016/j.gastha.2022.04.010

**Published:** 2022-04-25

**Authors:** Anish Vinit Patel, Mahmoud A. Ali

**Affiliations:** 1 Division of Gastroenterology & Hepatology, Rutgers Robert Wood Johnson Medical School, New Brunswick, New Jersey; 2 Department of Pathology & Laboratory Medicine, Rutgers Robert Wood Johnson Medical School, New Brunswick, New Jersey

A 61-year-old Caucasian man with tonsillar squamous cell carcinoma (SCC, stage T2N1, diagnosed 1 year ago) underwent an endoscopic percutaneous gastrostomy tube (PEG) placement for oropharyngeal dysphagia at the time of diagnosis. Given pulmonary metastases, he was begun on immunotherapy. Upper endoscopy was scheduled for PEG tube replacement. He denied abdominal pain, melena, and hematochezia. Hemoglobin was 11.6 g/dL. The upper endoscopy found a 3-cm ulcerated mass along the greater curvature of the gastric body (Figure A and Figure B). This was located 2 cm away from the internal PEG bumper. He underwent an uneventful PEG replacement. Biopsies demonstrated SCC (Figure C), with staining positive for p40 (Figure D). This established the diagnosis of gastric metastasis of tonsillar SCC. He was continued on immunotherapy.

Head and neck SCC metastasize to the lung, bone, and liver. We demonstrate here one of the few reported cases of tonsillar metastases to the stomach, which is one of the least common primary malignancies that result in gastric metastases. Although our patient did not have metastases at the gastric stoma, PEG tubes have been suggested as a risk factor for distal seeding of metastases. Gastroenterologists should consider direct PEG placement instead of pull-through placement in such patients.

**Figure:**
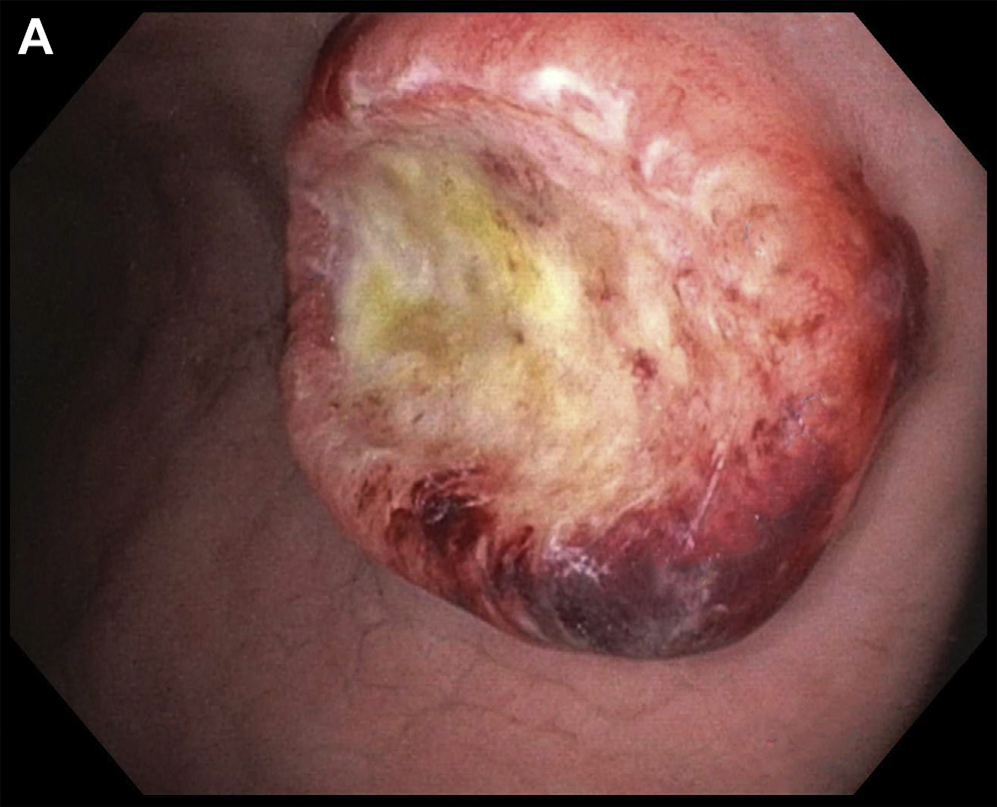

**Figure:**
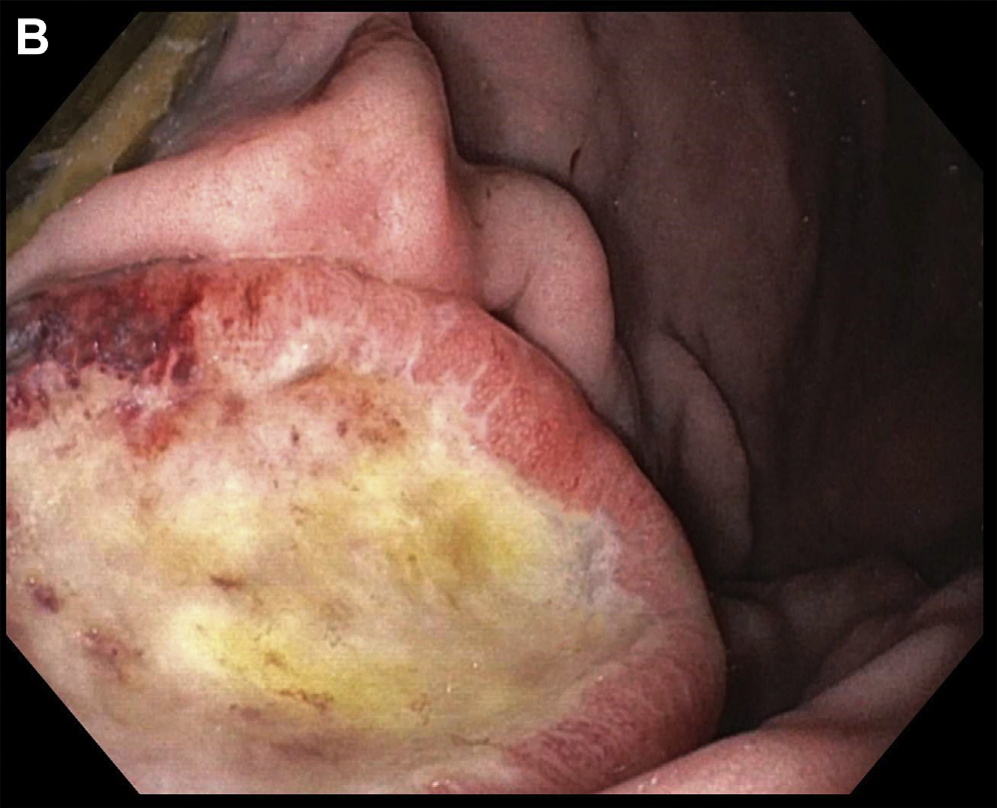

**Figure:**
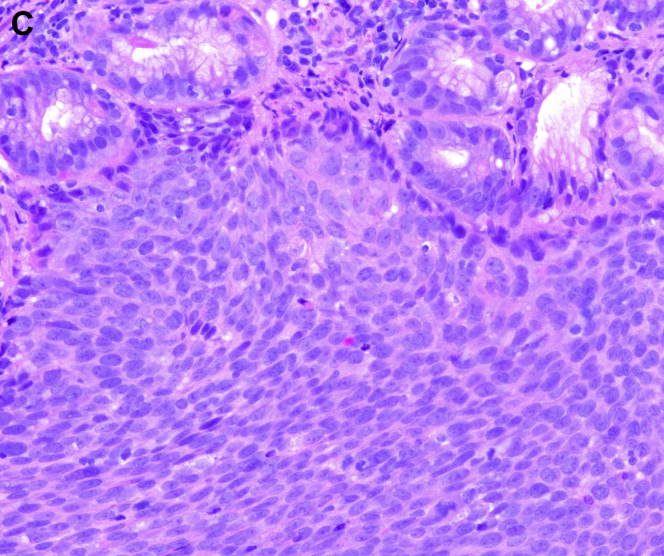

**Figure:**